# Small Particle Aerosol Exposure of African Green Monkeys to MERS-CoV as a Model for Highly Pathogenic Coronavirus Infection

**DOI:** 10.3201/eid2612.201664

**Published:** 2020-12

**Authors:** Allison Totura, Virginia Livingston, Ondraya Frick, David Dyer, Donald Nichols, Aysegul Nalca

**Affiliations:** Author affiliation: US Army Medical Research Institute of Infectious Diseases, Fort Detrick, Maryland, USA

**Keywords:** COVID-19, coronavirus disease, SARS-CoV-2, severe acute respiratory syndrome coronavirus 2, viruses, respiratory infections, zoonoses, MERS, MERS-CoV, Middle East respiratory syndrome, SARS-CoV, primate, model, medical countermeasures

## Abstract

Emerging coronaviruses are a global public health threat because of the potential for person-to-person transmission and high mortality rates. Middle East respiratory syndrome coronavirus (MERS-CoV) emerged in 2012, causing lethal respiratory disease in »35% of cases. Primate models of coronavirus disease are needed to support development of therapeutics, but few models exist that recapitulate severe disease. For initial development of a MERS-CoV primate model, 12 African green monkeys were exposed to 10^3^, 10^4^, or 10^5^ PFU target doses of aerosolized MERS-CoV. We observed a dose-dependent increase of respiratory disease signs, although all 12 monkeys survived for the 28-day duration of the study. This study describes dose-dependent effects of MERS-CoV infection of primates and uses a route of infection with potential relevance to MERS-CoV transmission. Aerosol exposure of African green monkeys might provide a platform approach for the development of primate models of novel coronavirus diseases.

Since 2002, three novel coronaviruses have emerged into human populations, causing severe respiratory disease: severe acute respiratory syndrome coronavirus (SARS-CoV) during 2002–2004; Middle East respiratory syndrome coronavirus (MERS-CoV), starting in 2012; and most recently, severe acute respiratory syndrome coronavirus 2 (SARS-CoV-2), starting in 2019 (*1*,*2*). All 3 of these highly pathogenic coronaviruses can cause lethal respiratory disease characterized by acute atypical pneumonia. Subclinical or asymptomatic infection has been reported for both MERS-CoV and SARS-CoV-2, but the actual number of asymptomatic infections and the pathogenesis of mild cases are not well understood (*3*,*4*). Onset of clinical disease from highly pathogenic coronaviruses typically follows an incubation period of 2–14 days, beginning as mild and nonspecific influenza-like illness including fever, fatigue, rhinorrhea, or dry cough. Many patients progress to symptoms of dyspnea and atypical pneumonia, often requiring hospitalization or supportive medical intervention, including ventilation.

*Coronaviridae* is a family of positive-sense, single-stranded RNA genome enveloped viruses that includes the genera alphacoronavirus, betacoronavirus, gammacoronavirus, and deltacoronavirus. Highly pathogenic coronaviruses, including SARS-CoV, MERS-CoV, and SARS-CoV-2 (all betacoronaviruses), likely emerged from bats, which are a diverse reservoir of alphacoronaviruses and betacoronaviruses (*5*–*8*). Cross-species transmission of MERS-CoV or similar zoonotic precursor viruses from bats to camels established an intermediate reservoir of MERS-CoV in dromedary camels (*9*). MERS-CoV replicates in the upper respiratory tract of camels, but camels demonstrate only mild disease signs, and a high percentage of camels are seropositive for MERS-CoV antibodies (*10*,*11*).

The MERS-CoV enzootic cycle within dromedary camels likely facilitates continued emergence in humans, where animal workers and healthcare workers are at risk for occupational exposure to MERS-CoV transmission (*12*,*13*). Sporadic MERS cases on the Arabian Peninsula continue to seed outbreaks primarily in Saudi Arabia with the potential for exported MERS cases by travelers to other regions. A major outbreak of MERS occurred in 2015 in South Korea, where a single case in a traveler returning from Saudi Arabia resulted in 186 cases and an additional »16,000 contacts were traced to prevent viral spread (*13*). Outbreaks of MERS since 2012 have resulted in a total of >2,500 cases of MERS, whereas 8,096 cases were identified in the SARS epidemic, and >6 million cases of coronavirus disease (COVID-19) have been confirmed globally as of June 1, 2020 (*14*–*16*). Transmission of highly pathogenic coronaviruses is likely complex and thus difficult to characterize. MERS-CoV infection in humans is thought to result from direct and indirect exposure to infected camels, consumption of contaminated camel products, or close contact with infected MERS patients. Respiratory droplets likely facilitate transmission of highly pathogenic coronaviruses, including MERS-CoV (*17*). However, unlike with SARS-CoV and SARS-CoV-2, MERS-CoV person-to-person transmission is somewhat limited and not often observed outside of households or healthcare settings. Within healthcare settings, aerosol-generating procedures are associated with increased risk for transmission of coronaviruses from infected patients to healthcare workers (*18*,*19*).

No proven antiviral therapies or vaccines exist for highly pathogenic coronaviruses. Current treatment regimens for MERS include supportive care and administration of general antiviral drugs. However, most medical countermeasures for MERS lack conclusive anti-coronavirus activity supported by robust in vitro and in vivo models of MERS-CoV infection. Nonhuman primate (NHP) models of SARS-CoV were initially pursued but were never characterized to the extent necessary to support therapeutic evaluation (*20*–*23*). In particular, platform approaches to developing animal models of highly pathogenic coronavirus infection have considerable value in that they could be rapidly applied to novel emerging viruses where medical countermeasures are needed.

Prior development of NHP models of MERS-CoV has been reported in the common marmoset (*Callithrix jacchus*) model and rhesus macaque (*Macaca mulatta*) model. Rhesus macaques experienced only mild, transient respiratory symptoms when infected with 10^6^–10^8^ PFU of MERS-CoV by either intratracheal route (IT) or multiple route (MR) (IT, intranasal [IN], oral, and ocular routes concurrently) (*24*,*25*). Common marmosets had onset of more severe MERS disease signs in other NHP experiments using similar doses and routes of exposure, but discrepancies have been reported in the marmoset model dependent on route of exposure (*26*–*28*). In rhesus macaque and marmoset models of MERS-CoV infection, endpoints for therapeutic testing are not well defined. A lack of robust primate models that replicate severe MERS disease observed in humans is a major obstacle to evaluation of medical countermeasure against MERS-CoV infection. Therefore, in this study we exposed African green monkeys (AGMs) to aerosolized MERS-CoV to determine whether an AGM model recapitulates severe MERS disease signs to establish a platform that is useful for medical countermeasure development.

## Methods

### Animals

Animal research was conducted at the United States Army Medical Research Institute of Infectious Diseases (USAMRIID). Twelve wild-caught adult AGMs (*Chlorocebus aethiops*) of Caribbean origin (estimated ages 7–12 years old, weighing 3.9–7.8 kg) were included on this study. Animals were acclimated in Biosafety Level 3 (BSL-3) containment laboratory animal rooms for 7 days before virus exposure and housed individually.

### Ethics Statement

These experiments and procedures were reviewed and approved by the USAMRIID Institutional Animal Care and Use Committee. All research was conducted in compliance with the US Department of Agriculture Animal Welfare Act and Public Health Service policy and other federal statutes and regulations relating to animals and experiments involving animals, and adheres to the principles stated in the Guide for the Care and Use of Laboratory Animals, National Research Council, 2011. The facility is fully accredited by the Association for Assessment and Accreditation of Laboratory Animal Care, International. The animals were provided food and water ad libitum and checked at least daily according to the protocol. All efforts were made to minimize painful procedures; the attending veterinarian was consulted regarding painful procedures, and animals were anesthetized before phlebotomy and virus exposure. Animals were humanely euthanized at the end of study under deep anesthesia in accordance with current American Veterinary Medical Association Guidelines on Euthanasia and USAMRIID standard operating procedures.

### Virus and Cells

The virus (MERS-CoV EMC/2012, NR-44260) was obtained through the BEI Resources Repository (https://www.niaid.nih.gov/research/bei-resources-repository) at the National Institutes of Health’s National Institute of Allergy and Infectious Diseases. Virus was sequence verified with 100% identity to MERS-CoV EMC/2012 (GenBank accession no. JX869059). MERS-CoV was amplified on Vero E6 cells. Supernatants from infected cells were collected and clarified by centrifugation. Plaque assay in Vero (CCL-81) cells was used to titrate the amount of virus in samples, as previously described (*29*). Neutral red was used to visualize plaques at 2–3 days after inoculation. We used 50% plaque-reduction neutralization titer assays (PRNT_50_) to titrate neutralizing response in AGM serum, as described previously (*30*). Plates were visualized with crystal violet to plaques at 3 days after inoculation.

### Aerosol Exposures

Each AGM was anesthetized by intramuscular injection of ketamine (8–12 mg/kg) and challenged by aerosol, as previously described (*31*). In brief, the respiratory function of each of the AGMs was measured by using the Buxco Large Animal Whole Body Plethysmography (Data Sciences International [DSI], https://www.datasci.com) before aerosol challenge. Aerosol procedures were conducted by using a 16-liter, airtight Lexan chamber assembled in a head-only configuration for individual AGM exposures in a class III biologic safety cabinet located inside a BSL-3 suite. The aerosol spray was generated using a Collison Nebulizer (CH Technologies, https://chtechusa.com) to produce a highly respirable aerosol (flow rate 7.5 + 0.1 L/minute). The system generates a target aerosol of 1–3 µm mass median aerodynamic diameter determined by aerodynamic particle sizer. Samples of the aerosol collected from the exposure chamber using an all-glass impinger during each exposure were assessed using a plaque assay. The exposure dose for each animal was calculated from the minute volume determined with a plexiglass whole body plethysmograph box using Buxco FinePointe software. The total volume of aerosol inhaled was determined by the exposure time required to deliver the estimated inhaled dose (*31*).

### Animal Observation and Endpoint Criteria

AGMs were observed at least twice a day after aerosol exposure and scored for clinical signs of disease before (and while under) anesthesia. Any observable disease signs, including dehydration, lymphadenopathy, and respiratory signs, were recorded during physical examinations. Other observations such as biscuit and fruit consumption, condition of stool, and urine output were also documented, if possible. The early endpoint criteria for humane euthanasia were a >4 responsiveness score, or a >4°C decrease from baseline body temperature without anesthesia, or agonal breathing (Appendix).

### Telemetry

AGMs were implanted subcutaneously with a radiotelemetry device (DSI) >14 days before aerosol exposure. Body temperatures were recorded every 15 minutes using the DataQuest A.R.T. 4.1 system (DSI). Fever was predefined as 2 SDs above individual baseline temperature as determined by autoregressive integrated moving average modeling. Baselines for each animal were calculated by averaging the recorded 15-minute temperature intervals for 3 days before challenge.

### Sample Collection

**Figure 1:**
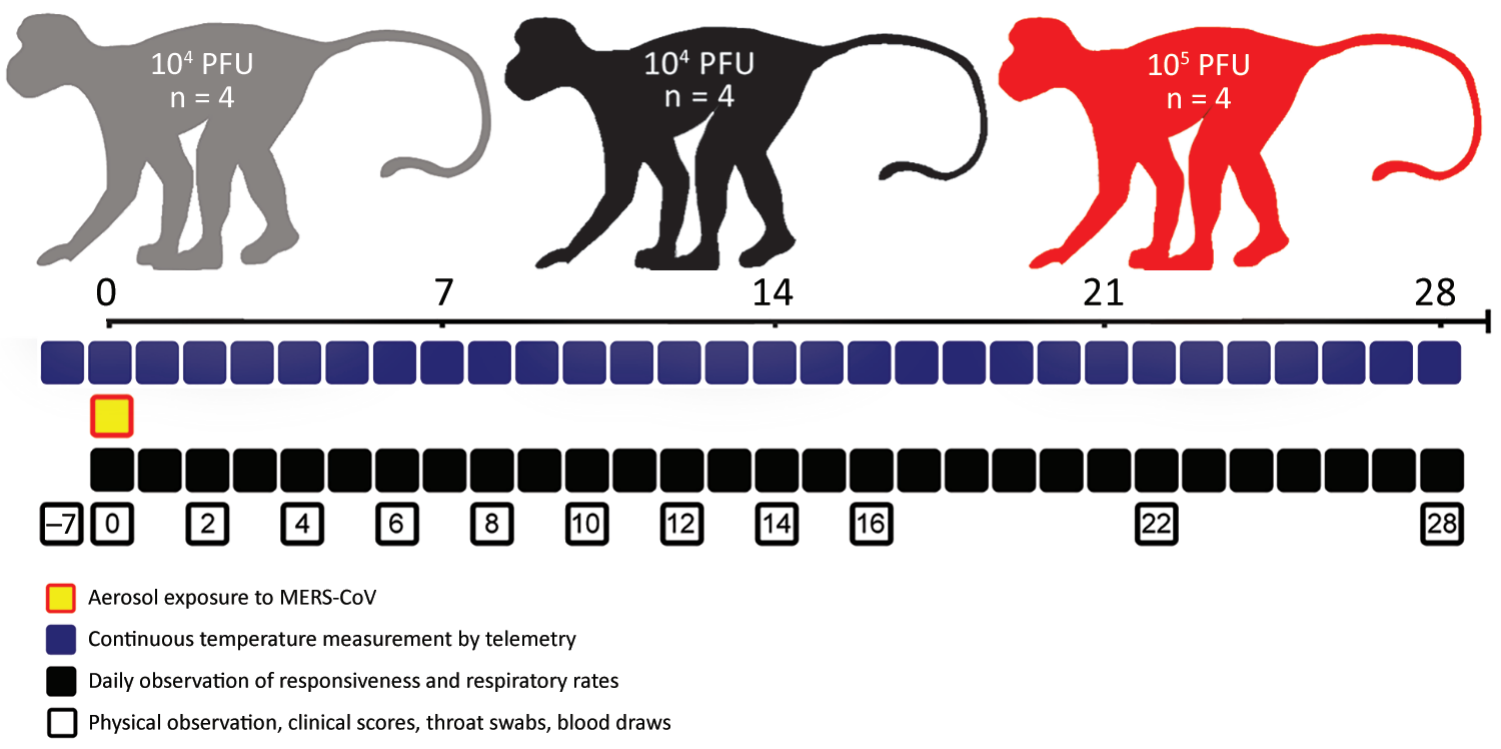
Study schedule for small particle aerosol infection of African green monkeys (AGMs) with MERS-CoV. Three groups of AGMs (4 in each group) were exposed to 3 different target doses of MERS-CoV EMC/2012 strain by small particle aerosol exposure. AGMs were observed at indicated days postinfection (shown by squares) for metrics that would indicate recapitulation of MERS-CoV infection in humans. MERS-CoV, Middle East respiratory syndrome coronavirus.

Blood samples and throat swab specimens were collected at indicated days after aerosol exposure to MERS-CoV (Figure 1). Puritan 6-inch 25–800–1PD sterile swabs (https://www.puritanmedproducts.com) were used for collection and were placed in 1-mL virus growth medium and frozen until further processing. Blood samples were collected from the femoral vein of AGMs anesthetized with 3 mg/kg intramuscular ketamine. Samples collected 7 days before exposure served as a reference baseline for each animal. Blood chemistry values were analyzed with VITROS 250 chemistry analyzers (Ortho Clinical Diagnostics, https://www.orthoclinicaldiagnostics.com).

### Necropsy and Histology

At the conclusion of this study (day 28 postinfection), each AGM was euthanized with an overdose of pentobarbitol and then submitted for necropsy. The necropsies were performed under BSL-3 biocontainment by a pathologist certified by the American College of Veterinary Pathologists. The samples for histology were fixed by immersion in containers containing 10% neutral-buffered formalin. These containers were held for >30 days under BSL-3 biocontainment before being transferred to the USAMRIID histology laboratory. The formalin-fixed tissues were then trimmed, processed, and embedded in paraffin according to standard operating procedures. The paraffin-embedded tissues were cut into sections 5 µ thick, which were placed onto glass microscope slides, stained with hematoxylin and eosin, and coverslipped before histologic evaluation by the study pathologist.

## Results

### Clinical Disease Signs Observed in AGMs Exposed to Aerosolized MERS-CoV

**Figure 2:**
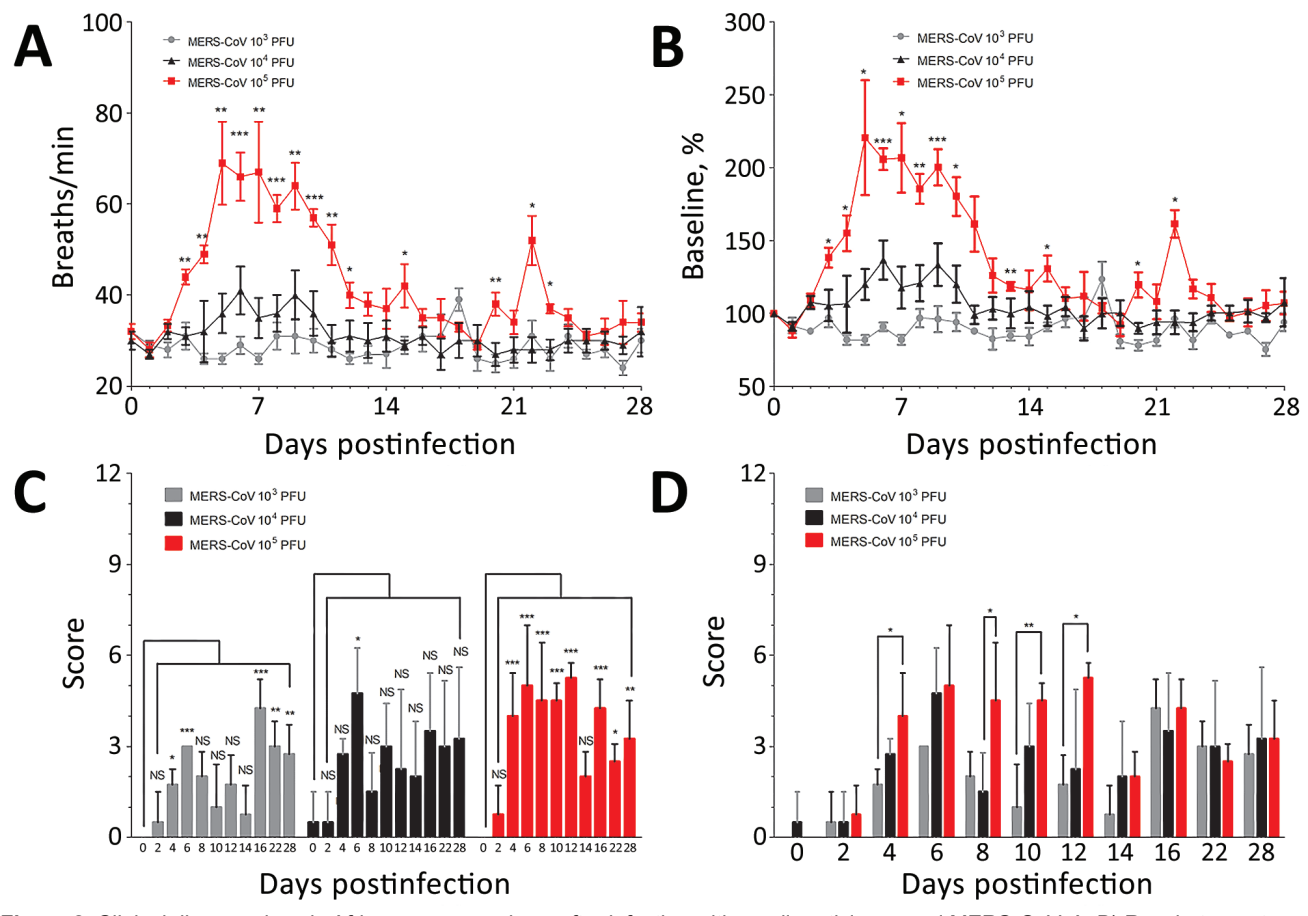
Clinical disease signs in African green monkeys after infection with small particle aerosol MERS-CoV. A, B) Respiratory rates were observed twice daily for all groups and recorded. Statistically significant differences on the graph reflects analysis comparing respiratory rates between the 10^3^ and 10^5^ PFU dose groups. C, D) Clinical scores incorporated signs of MERS-CoV infection, based in part on observations of responsiveness, respiratory function (other than respiratory rate), lymphadenopathy, and dehydration. Differences between respiratory rate and clinical score groups were determined by using 1-way analysis of variance (Tukey’s multiple comparison test; *p<0.05, **p<0.01, ***p<0.001). MERS-CoV, Middle East respiratory syndrome coronavirus.

To enable comparison of aerosol exposure to previously published NHP model development studies, AGMs were exposed to aerosolized MERS-CoV strain EMC/2012, which is the same strain used previously by other research groups (*24*,*26*,*27*). The expected range of doses was 10^3^–10^5^ PFU. The actual range of the infection was 10^2.88^–10^4.57^ PFU (Table 1). All of the AGMs survived the MERS-CoV exposure and subsequent manipulations to the conclusion of the study at 28 days postinfection (Figure 2). Of note, the highest dose group of AGMs exposed to aerosolized MERS-CoV in this study were estimated to have received considerably lower doses than in prior published NHP models using MR (5–7 × 10^6^ 50% tissue culture infectious dose) or IT only (5 × 10^6^ – 5 × 10^7^ PFU) routes of infection (*24*–*27*).

**Table 1:** Inhaled doses of African green monkeys exposed to MERS-CoV*

Animal ID	Sex	Target dose, 10^x^ PFU	Actual dose, PFU	Actual dose, 10^x^ PFU
1	F	3	7.60 × 10^2^	2.88
2	M	3	1.17 × 10^3^	3.07
3	M	3	8.70 × 10^2^	2.94
4	F	3	1.11 × 10^3^	3.04
5	F	4	6.31 × 10^3^	3.80
6	M	4	7.79 × 10^3^	3.89
7	M	4	7.25 × 10^3^	3.86
8	F	4	4.65 × 10^3^	3.67
9	F	5	2.78 × 10^4^	4.44
10	M	5	3.75 × 10^4^	4.57
11	M	5	2.83 × 10^4^	4.45
12	F	5	2.27 × 10^4^	4.36

*MERS-CoV, Middle East respiratory syndrome coronavirus.

To determine whether exposure of AGMs to aerosolized MERS-CoV results in observable disease signs that recapitulate MERS disease observed in human cases, physical observations of AGMs were performed, including calculation of respiratory rates (Figure 2, panels A, B) and clinical scores (Figure 2, panels B–D). Clinical scores included observation of disease signs, including responsiveness, lymphadenopathy, dehydration signs, and respiratory signs by physical examination. Clinical disease signs that recapitulated human cases of MERS were observed in all groups but were most pronounced in the group that received the highest dose of MERS-CoV (Figure 1, panels C, D). All groups had increased clinical scores over the course of infection compared with pre-infection clinical scoring. No statistically significant changes in weight of AGMs were observed over the course of the study or between groups (data not shown). The 10^5^ PFU group had significantly higher respiratory rates than the 10^3^ PFU group, beginning at 3 days postinfection (dpi) and continuing through 10 dpi (Figure 2, panels A and B). Respiratory rates were not significantly different between the 10^4^ PFU dose group and 10^3^ PFU dose group. Significantly higher clinical scores were observed in the 10^5^ PFU group of AGMs than the 10^4^ PFU or 10^3^ PFU group (Figure 2, panel D). Onset of respiratory disease signs occurred at 6 dpi and persisted in some animals through 16 dpi. All of the AGMs in the 10^5^ PFU group displayed observable respiratory disease signs, including chest congestion, rales, or wheezing. Baseline temperatures were measured over a 24-hour period before infection with aerosolized MERS-CoV. Individual AGMs in 10^4^ and 10^5^ PFU dose group (2 of 4 AGMS in each group) had elevated temperature after exposure to MERS-CoV aerosols during 1–3 dpi but were not febrile (Appendix Figure 1).

### Viral Replication and Tissue Damage Resulting from MERS-CoV Infection of AGMs

**Figure 3:**
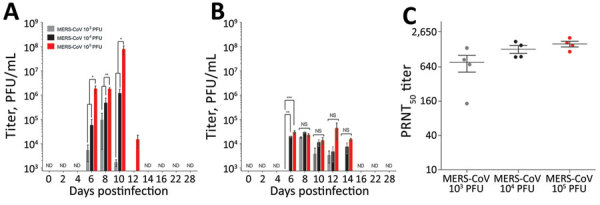
Detection of MERS-CoV viral loads in throat swabs and serum from African green monkeys (AGMs). A, B) Infectious MERS-CoV was titrated on Vero cells from throat swab samples (A) and serum samples (B) collected at indicated days after MERS-CoV aerosol exposure of AGMs. C) PRNT_50_ titers were assessed for AGM serum samples collected at 28 days postinfection. Plaque assays and PRNT_50_ assays were completed for each sample in triplicate. Differences between groups were evaluated by using 1-way analysis of variance (Tukey’s multiple comparison test; *p<0.05, **p<0.01, ***p<0.001). MERS-CoV, Middle East respiratory syndrome coronavirus; ND, not detected; NS, not significant; PRNT_50_, 50% reduction plaque reduction neutralization test.

To determine whether respiratory disease signs observed in AGMs were the result of robust MERS-CoV viral replication, viral loads were titrated from throat swab specimens and serum samples. Virus was detected by plaque assay in throat swab specimens collected 6 dpi from all AGMs after exposure to aerosolized MERS-CoV. Significantly higher viral titers were observed in the throat swab specimens collected from the highest dose group (10^5^ PFU) of AGMs than the lower 2 dose groups during 6–12 dpi (Figure 3, panel A). Onset of respiratory symptoms recorded during physical evaluations of AGMs was concurrent with detection of virus in throat swab specimens. Significantly higher viral titers in the serum were observed in the 10^5^ PFU group and 10^4^ PFU group compared with the 10^3^ PFU group in the serum at 6 dpi (Figure 3, panel B). At the conclusion of the study, all of the AGMs had measurable neutralizing antibody titers in the serum, although no statistically significant difference was observed in neutralizing titers between the dose groups (Figure 3, panel C). Elevated blood enzyme chemistry values were observed in AGMs infected with the highest dose of MERS-CoV (Appendix Figure 2). No statistically significant differences were observed in blood urea nitrogen or creatinine levels (Appendix Figure 2, panel A–D). Significantly elevated levels of enzymes indicative of liver damage, aspartate aminotransferase and gamma-glutamyl transferase, were observed in the 10^5^ PFU group at 8 dpi and 10 dpi (Appendix Figure 2, panels E–H).

**Figure 4:**
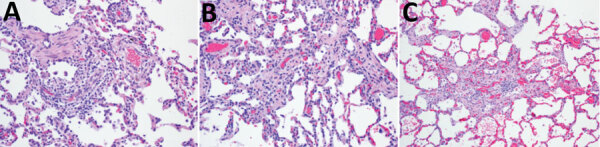
Interstitial pneumonia observed in lung samples from African green monkeys. Hemolysin and eosin–stained lung sample sections were collected from African green monkeys exposed to aerosolized Middle East respiratory syndrome coronavirus at 28 days postinfection. Images shown reflect a sample of lung disease observed in the 10^3^ PFU dose group (A) (original magnification × 20), 10^4^ PFU dose group (B) (original magnification × 20), and 10^5^ PFU dose group (C) (original magnification × 10). Samples were evaluated by a trained pathologist and scored for lesions associated with severe respiratory disease.

Although the AGMs survived the infection, all of the AGMs exposed to aerosolized MERS-CoV showed mild or minimal lung lesions at 28 dpi (Table 2; Figure 4). At the point in the disease course when lung samples were collected, all of the AGMs were recovering from the infection, although some were still experiencing very mild disease signs correlated by the clinical disease score (Figure 2, panel C). The most common histopathologic observation was multifocal interstitial pneumonia, which was likely attributable to the MERS-CoV aerosol exposure. The location, severity, and type of lung lesion observed appeared to be independent of the dose of aerosolized MERS-CoV. Another common finding was lymphoid hyperplasia in the mediastinal lymph nodes, the tracheobronchial lymph nodes, or both, which can be attributed to antigenic stimulation caused by the viral infection (data not shown). These observations were not clinically significant and, because these lymph nodes receive lymphatic drainage from the lungs, this finding was not unexpected after an aerosol exposure to a virus. Tissues from other major organs were surveyed for pathologic disease signs, but no other findings were consistent with MERS disease (data not shown).

**Table 2:** Summary of histological findings from African green monkeys exposed to MERS-CoV*

Animal ID	Sex	Target dose, 10^x^ PFU	Type of lung lesion	Location and severity of lung lesion			
				Left superior	Right superior	Left inferior	Right inferior
1	F	3	Multifocal interstitial pneumonia	Minimal	Minimal	Minimal	Minimal
2	M	3	Multifocal interstitial pneumonia	Minimal	Minimal	NLP	NLP
3	M	3	Multifocal broncho-interstitial pneumonia	Mild	Mild	Minimal	Minimal
4	F	3	Multifocal interstitial pneumonia	Minimal	Minimal	Minimal	Minimal
5	F	4	Multifocal broncho-interstitial pneumonia	Minimal	Minimal	NLP	NLP
6	M	4	Multifocal foreign-body bronchiolitis	NLP	NLP	Mild	Mild
7	M	4	Multifocal broncho-interstitial pneumonia	Minimal	Minimal	NLP	NLP
8	F	4	Multifocal interstitial pneumonia	Minimal	Minimal	Mild	Mild
9	F	5	Multifocal interstitial pneumonia	NLP	NLP	Minimal	Minimal
10	M	5	Multifocal interstitial pneumonia	Mild	Mild	Minimal	Minimal
11	M	5	Multifocal interstitial pneumonia	Minimal	Minimal	Minimal	Minimal
12	F	5	Multifocal interstitial fibrosis	NLP	NLP	Minimal	NLP

*MERS-CoV, Middle East respiratory syndrome coronavirus; NLP, no lesion present.

## Discussion

Currently, the world is experiencing a pandemic of a novel coronavirus (SARS-CoV-2). More COVID-19 cases have occurred in the first month since identification than all of the previous SARS-CoV and MERS-CoV epidemics combined. Novel coronaviruses from the *Betacoronavirus* genus have emerged into the human population 3 separate times in <20 years: SARS-CoV (2002), MERS-CoV (2012), and SARS-CoV-2 (2019). In each instance, the novel coronavirus most likely emerged from viruses originally circulating in bats (*5*,*7*,*8*). During the emergence of SARS-CoV and MERS-CoV, spillover into human populations was driven by an intermediate animal reservoir in closer proximity to humans (civets or raccoon dogs for SARS-CoV and dromedary camels for MERS-CoV) (*9*,*31*). Whether SARS-CoV-2 emergence resulted directly through transmission from bats to humans or through another intermediate animal host remains unknown. Human disease from emergent coronaviruses manifested as a respiratory syndrome with the hallmarks of atypical pneumonia, progressing to more severe lung dysfunction accompanied by varying degrees of illness (asymptomatic illness to long-term lung dysfunction) and even death (»10% mortality rate from SARS, »35% from MERS, and yet to be determined from COVID-19) (*32*). Forewarned by the knowledge that coronaviruses circulating in animals can cause severe disease in humans, developing platform approaches for the generation of animal models is needed to allow evaluation of medical countermeasures against coronaviruses. The development of well-characterized NHP models of coronavirus infection could provide a complementary approach to small animal models of pathogenesis, transmission, and countermeasure development.

In this study, we initiated development of an AGM model of highly pathogenic coronavirus infection by aerosol as a first step to establishing a platform for medical countermeasure testing against highly pathogenic coronaviruses. A prior study in NHP models of SARS-CoV delivered by combined IN/IT route compared disease signs in multiple NHP species (e.g., rhesus macaques, cynomolgus macaques [*Macaca fascicularis*], and AGMs) (*21*). Although none of the NHPs had onset of severe disease signs from SARS-CoV infection, AGMs had higher titers of virus recovered from nasal swabs and throat lavage than rhesus or cynomolgus macaques, particularly at later times postinfection (4–10 dpi). Building on this observation, we evaluated 3 different target doses of MERS-CoV delivered by aerosol to determine if AGMs have a dose-dependent disease response to MERS-CoV infection. Although we did not observe lethal or severe MERS disease signs, all of the AGMs in the highest dose group had clinical disease signs and elevated respiratory rates.

Previous studies of MERS-CoV in NHPs focused on infection of either rhesus macaques or marmosets. The approaches of different research groups typically used very high titer inoculum delivered by either MR or IT-only routes of infection. Rhesus macaques were observed to have only very mild disease signs (if any) after MERS-CoV infection by either MR or IT route (*24*,*25*). A study of a small particle aerosol exposure of rhesus macaques to MERS-CoV yielded subclinical infection with no disease signs (*33*). Viral replication of MERS-CoV in rhesus macaques is limited and difficult to detect, indicating limitations for the use of this model for evaluation of medical countermeasures to MERS-CoV infection. Marmosets infected with MERS-CoV were observed to have more severe disease signs by MR but not the IT route (*24*–*27*). Marmosets infected by MR had high viral loads in serum and throat swab specimens (*26*), as was observed in this study with AGMs infected by the aerosol route. Although the marmoset model of MERS-CoV infection by MR yields severe respiratory disease, marmosets are a smaller NHP species that might present challenges for researchers evaluating medical countermeasures because of sampling limitations. NHP model development in rhesus and marmoset models used a higher titer inoculum of MERS-CoV than was used in this study of aerosol exposure of AGMs to MERS-CoV and did not compare dose-response. The response of animal models to differing doses of coronavirus infection might play an important role in the evaluation of viral pathogenesis of subclinical disease compared with more severe disease.

Additional refinements to our AGM model of MERS-CoV infection are needed for medical countermeasure testing and evaluation. Although the AGMs had observable disease signs, including elevated respiratory rates and other respiratory disease signs, our model did not recapitulate severe disease or lethality as observed in MERS patients. However, we are encouraged by the dose dependence of the clinical disease signs and response to MERS-CoV infection that we observed, which indicates the potential for increased severity of disease in future iterations of this study using higher titers aerosol exposure. In addition, other NHP studies of MERS-CoV model development used early endpoint euthanasia to explore pathologic outcomes at acute times during viral infection that we would like to compare in future studies. The presence of pneumonia in all of the AGMs at the end of the study indicates that it is likely that pathologic findings related to MERS disease might be present during the acute phase of infection when respiratory disease signs are observed. In addition, the presence of histologic lesions even after AGMs had mostly recovered from MERS-CoV infection might be of use as a model of long-term lung dysfunction observed in MERS and SARS survivors.

In conclusion, animal models of viral infection must couple methods with desired outcomes; routes and doses of infection must be sufficiently reproducible, but they must also target the viral inoculum to the appropriate tissues to model respiratory disease. Recent efforts to develop an NHP model of SARS-CoV-2 infection for evaluation of vaccines and other medical countermeasures have focused on infection of rhesus macaques or cynomolgus macaques by IN/IT routes or MR with varying respiratory disease signs (*34*–*36*). Animal model development, particularly where it applies to generating a model that is appropriate for medical countermeasure evaluation has been understudied in coronavirus research. We anticipate that the approach we have defined in this study can provide a starting point for additional model development in AGMs or other NHPs using the aerosol route of infection for MERS-CoV, SARS-CoV, SARS-CoV-2, or other novel highly pathogenic coronaviruses.

Appendix Additional information about small particle aerosol exposure of African green monkeys to MERS-CoV as a model for highly pathogenic coronavirus infection.
