# Supplementary material for: Small Particle Aerosol Exposure of African Green Monkeys to MERS-CoV as a Model for Highly Pathogenic Coronavirus Infection
Source: Emerg Infect Dis. 2020 Dec;26(12):2835–43. doi: 10.3201/eid2612.201664 (PMC7706928; doi:10.3201/eid2612.201664)
Supplement: Appendix — Additional information about small particle aerosol exposure of African green monkeys to MERS-CoV as a model for highly pathogenic coronavirus infection. [file 20-1664-Techapp-s1.pdf]

Article DOI: https://doi.org/10.3201/eid2612.201664

Small Particle Aerosol Exposure of African Green Monkeys to MERS-CoV as a Model for Highly Pathogenic Coronavirus Infection

Appendix

Appendix Table. Clinical scoring parameters

Parameter (type of assessment)	Description	Score
Visual observations		
Responsiveness	Active (normal species specific behavior)	0
	Slightly diminished general activity	1
	Mildly unresponsive (becomes active when approached), occasional prostration	2
	Moderate unresponsiveness (may require prodding to respond), weakness	3
	Moderate to severe unresponsiveness requires prodding, moderate prostration	4
	Moribund, severe unresponsiveness, pronounced prostration	5
Respiratory function (visual)	Normal - no apparent changes in respiration	0
	Mildly labored respiration	1
	Moderately labored respiration	2
	Agonal breathing	3
Physical observations		
Respiratory function (tactile/auditory)	Normal (no apparent changes in respiration)	0
	Mildly labored respiration	1
	Moderately labored respiration	2
	Severely labored respiration	3
Lymphadenopathy (palpation)	Normal (no apparent changes to lymph nodes)	0
	Enlargement of lymph nodes <5 mm	1
	Enlargement of lymph nodes 5–10 mm	2
	Enlargement of lymph nodes >10 mm	3
Dehydration (skin turgor)	Normal (no apparent changes to skin turgor)	0
	Mild dehydration	1
	Moderate dehydration	2
	Severe dehydration	3

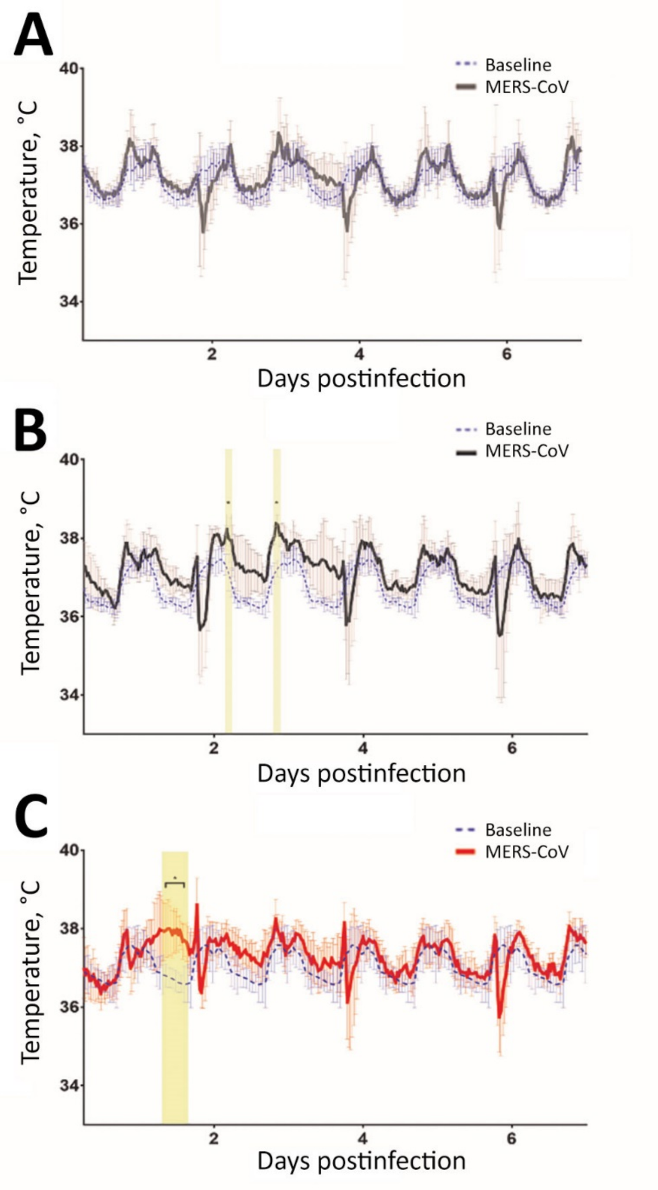

Appendix Figure 1. Body temperature in AGMs following MERS-CoV aerosol exposure. Baseline temperatures were measured over a 24 hour period before infection with aerosolized MERS-CoV (blue dashed lines) and following exposure to MERS-CoV (solid lines) for groups of AGMs exposed to 10^3 PFU doses (A) shown by gray lines, 10^4 PFU doses (B) shown by black lines, or 10^5 PFU doses (C) shown by red lines. Administration of anesthesia for physical observation and clinical scoring of AGMs corresponded with a brief drop in temperature (2, 4, 6 dpi). Elevated temperature for groups of AGMs was defined as 1.5 degrees above baseline. Differences between groups of AGMs were determined by comparing average experimental temperature with average baseline temperature by paired *t*-test (*, yellow shaded regions). Although groups of NHPs had brief periods of elevated temperature following MERS-CoV exposure, individual animals did not meet study criteria for fever as defined in the experimental methods.

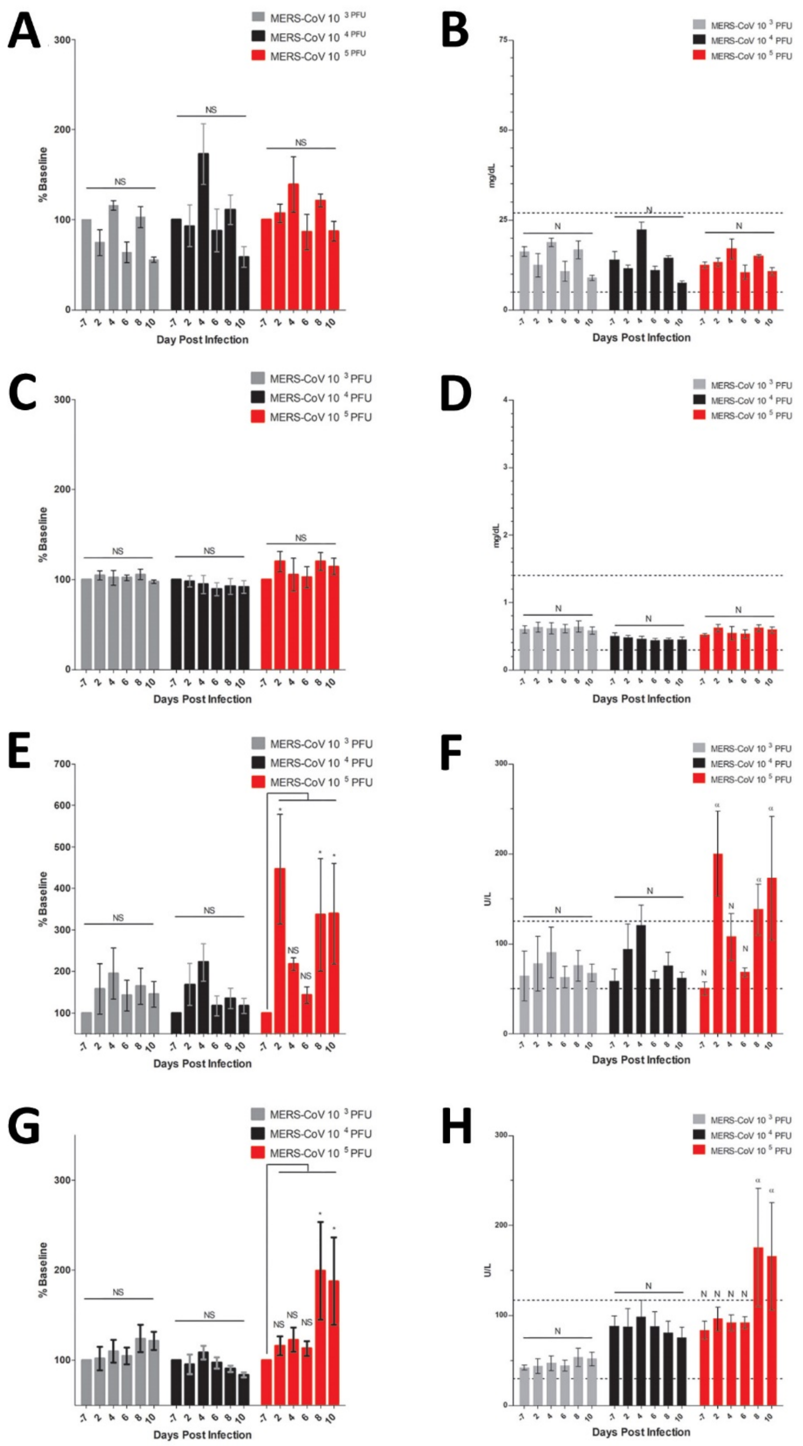

Appendix Figure 2. Elevation of enzymes from AGM serum following MERS-CoV aerosol exposure. Serum was collected for blood chemistry analysis of BUN (A,B), CRE (C,D), AST (E,F), and GGT (G,H). Elevation of serum enzymes was determined to be statistically significant by comparing to pre-exposure serum values by the Mann-Whitney test (*p<0.05) (A,C,E,G) and if mean values were above established normal values (α, above normal range) for healthy AGMs (B,D,F,H).
